# Kyasanur Forest Disease: An Epidemiological Investigation and Case-Control Study in Shivamogga, Karnataka, India-2022

**DOI:** 10.3389/ijph.2024.1606715

**Published:** 2024-10-18

**Authors:** Srividya K. Vedachalam, Bhavesh L. Rajput, Sushma Choudhary, Darshan Narayanaswamy, Sharath Chandra, Pallavi D. M., Padma M. Rajagopal, Tanzin Dikid

**Affiliations:** ^1^ Department of Epidemiology, National Centre for Disease Control (NCDC), Delhi, India; ^2^ South Asia Field Epidemiology and Technology Network, New Delhi, India; ^3^ Viral Diagnostic Laboratory, Department of Health and Family Welfare Services, Shivamogga, India; ^4^ Integrated Disease Surveillance Programme, State Surveillance Unit, Bangalare, India; ^5^ State Surveillance Unit, Bangalore, India

**Keywords:** arbovirus infections, viral zoonoses, tick-borne encephalitis, risk factors, vaccination

## Abstract

**Objective:**

Kyasanur Forest Disease (KFD) is a viral zoonosis reported from Karnataka, India. We investigated cases in the Shivamogga district, Karnataka, to describe the epidemiology and identify risk factors in the affected block in 2022.

**Methods:**

A case was defined as a laboratory-confirmed KFD-positive resident of Shivamogga from 1 January-31 May 2022. We extracted the records of KFD cases from district surveillance. We conducted a 1:3 case-control study in the Thirthahalli block. We enrolled laboratory-confirmed KFD-positive Thirthahalli residents from January to May 2022 as cases, and residents without “fever with myalgia” as controls. We reported adjusted odds ratios (aOR) with 95% confidence intervals (CI).

**Results:**

Shivamogga reported 35 cases, with a median age of 46 (4–75) years, of which 51% were men, and one death. Among 25 cases and 90 controls, knowledge of avoiding recent monkey death sites was low (cases = 0%, controls = 11%). Monkey death sites within 500 m [aOR = 8.6 (1.8–41.9)] and household tick exposure [aOR = 3.7 (1.3–10.7)] were independent risk factors.

**Conclusion:**

This was a laboratory-confirmed cluster of KFD cases in Thirthahalli, with residence near a monkey death site and household tick exposure considered significant risk factors. We recommend evaluating monkey carcass disposal procedures and increasing awareness of tick protective measures.

## Introduction

Kyasanur Forest Disease (KFD) is a tick-borne viral hemorrhagic fever, spread by infected *Haemaphysalis spinigera* ticks, that first reported in 1957 from the Kyasanur forest in the Shivamogga district, Karnataka state, India [1]. Monkeys, rodents and shrews are the common animal hosts of the KFD virus with epizootics in primates resulting in high mortality. The death of a monkey is considered a sentinel event as it is an early sign of disease transmission and can predict a possible epidemic in the area [2]. A dead monkey infected with KFD represents a hotspot for the spread of disease because infected ticks fall off the monkey carcass and can in turn infect other mammals and humans in the immediate area [3]. In humans, the disease causes an acute febrile illness with myalgia, prostration, headache, nausea and bleeding, after 3–8 days of exposure, with a reported case mortality rate of 3%–10% [2]. The KFD prevention and control program of the state of Karnataka in the affected areas includes surveillance of human cases, monkey deaths, mapping of tick pool positivity, followed by KFD vaccination in humans and vector control in cattle. The KFD vaccine is a formalin inactivated vaccine, targeted at individuals 6 years of age and older, with two primary doses 1 month apart, a booster dose after 6–9 months after the second primary dose and an annual booster in the at-risk population. The KFD program defines at-risk population as people living within 5 km of a either a laboratory-confirmed KFD-positive human case, or a tick-pool positive area or a monkey death site [4]. Despite these measures, KFD outbreaks have been regularly reported in Karnataka annually since 1957, with the Shivamogga district reporting a consistently high burden of 3,336 cases reported since 1957 [5, 6]. Recently, outbreaks have also been reported from the previously unaffected district of Karnataka and the neighboring states of Tamil Nadu, Kerala, Goa and Maharashtra [3, 7, 8].

Starting in January 2022, the Shivamogga district reported a rise in KFD cases, with cases in Thirthahalli taluk (sub-district), including one death in May 2022. A team consisting of two Epidemic Intelligence Service officers from the National Centre for Disease Control, New Delhi (NCDC), consultants from the Karnataka State Surveillance Unit and Viral Diagnostics Laboratory (VDL), a state entomologist, and field workers involved in KFD surveillance was deputed to investigate the cases. We investigated the cases reported in the Shivamogga district, including the cluster reported in Thirthahalli to describe their epidemiology. We reviewed the KFD prevention and control program records within the Thirthahalli block to assess relevant aspects of program implementation. We conducted a case-control study in Thirthahalli to identify risk factors that put the population of a selected taluk at risk of contracting KFD.

## Methods

### Time Trend Analysis and Outbreak Verification

We obtained the surveillance list of laboratory-confirmed KFD cases, either immunoglobulin M Enzyme-Linked Immunosorbent Assay (IgM-ELISA) or reverse transcription polymerase chain reaction (RT-PCR) for the Shivamogga district from January 2018 to May 2022 from the district surveillance unit, Shivamogga to calculate the time-trend of cases and verify the existence of an outbreak.

### Descriptive Epidemiology and Hypothesis Generation

#### Case Definition for Descriptive Epidemiology

We defined a case as an IgM ELISA or RT-PCR confirmed KFDV-positive resident of the Shivamogga district from 1 January to 31 May 2022. We analyzed the list of KFD cases from the Shivamogga district between 1 January and 31 May 2022 to characterize their descriptive epidemiology.

#### Study Setting

We used a clinical abstraction tool to extract clinical data of cases admitted to the taluk (secondary care) hospital, Thirthahalli and Manipal hospital, Karnataka (tertiary care) from January to May 2022 to describe the clinical course of the hospital. We selected these hospitals as these are the only government-runsecondary and tertiary care referral healthcare facilities serving the area. Based on the literature review and data gathered from descriptive epidemiology, we hypothesized that not being vaccinated against KFD was associated with higher odds of acquiring KFD. We selected the taluk reporting the highest number of laboratory-confirmed KFD cases from 1 January to 31 May 2022 to conduct an unmatched case-control study to identify risk factors for contracting KFD.

### Case Control Study

#### Case and Control Definitions

For the case-control study, a case was defined as an IgM ELISA or RT-PCR confirmed KFDV positive resident of Thirthahalli taluk from 1 January to 31 May 2022. A control was defined as a resident of Thirthahalli taluk for at least 90 days during the period from 1 January to 31 May 2022 and who did not develop symptoms of fever and myalgia during the period of stay.

#### Sample Size Calculation

A sample size of 100 (cases 25: controls 75) was calculated using stat calc (Epi Info V7.2.5.0) with a 95% confidence interval, 80% power, and a 1:3 case to control ratio using “non-vaccination for KFD” as exposure considering 83% of cases and 52% of controls exposed [9].

We identified villages in Thirthahalli taluk that had a confirmed KFD case-patient from January to May 2022 (case village). We contacted all cases present in the line list by telephone, making up to three attempts to contact each case. All available cases willing to be interviewed were included and in-person interviews were conducted where feasible. To select controls, a Google map of the area showing the case villages was obtained and an equal number of villages (control villages) were identified surrounding these case villages that did not have a case in 2022. To select controls, house numbers were obtained from the village health worker, and one household from each case village and two households from each control village were selected randomly using a random number generator application on a mobile phone. Only one person from each household, whose birth month was closest to the interview month (June) and who was willing to be interviewed, was selected as a control to ensure randomization in the selection of controls.

### Assessment of the KFD Prevention and Control Program

We reviewed program operational guidelines, district vaccination coverage records, monkey death and tick pool surveillance records and time to diagnosis and admission for confirmed cases in Thirthahalli from August 2021 to May 2022 to evaluate prevention and control efforts. We categorized each activity conducted by the program as “satisfactory” if it was done according to the program’s operational guidelines and “unsatisfactory” otherwise [4]. We interviewed relevant state and district officials to understand implementation challenges.

### Data Collection Tool

We interviewed cases and controls using a pre-tested structured data collection tool. We collected data on socio-demographic variables; exposure history (including contact with ticks/ dry leaves during farm/ forest foraging, and proximity of respondent’s house to monkey death sites); knowledge of disease acquisition and prevention; vaccine doses received and clinical history (only from cases). Data on risk factors such as house location (near forest/plantation), house surroundings (with dry leaf heap, firewood stacks, bushes), and presence of cattle were determined by the interviewer from an observation checklist during the site visit. Vaccination details were verified from the vaccination records at the community health center and district laboratory. Data were collected in Epi Collect 5 and analyzed using Epi-info version 7.2.5.0.

### Data Processing and Analysis

We dichotomized responses to analyze exposures related to house distance from monkey death sites (house <500 m from the monkey death site vs. >500 m) and house distance from forest edge (house <50 m from the forest edge vs. >50 m). We created two composite variables: household tick exposure and low socioeconomic status by combining responses from similar classes of exposures. We combined responses to tick sightings AND storage of dry leaves/firewood in the house to create the variable household tick exposure (yes vs. no); if a respondent said yes to questions related to tick sightings at home in the previous 3 months and storage of dry leaves/firewood in the house, then responses were coded as yes, otherwise as no. For low socioeconomic status, we combined responses from respondents with no formal education OR residing in a non-cemented house OR not owning cattle; if the respondent answered yes to any of these, then responses were coded as yes, otherwise as no. Questions on knowledge about disease acquisition and prevention were framed as multiple-choice questions with the opportunity to choose more than one option.

We calculated medians and proportions to summarize the results. We described cases by time, place and person. We compared proportions using χ2 and tested statistical significance at p < 0.05. We used a Chi-square test to compare the socio-demographic characteristics of cases and controls considering a p-value <0.05 as significant. We dichotomized exposures and conducted univariate analyses for cases and controls. Exposures previously described as significant in the literature (involved in handling or storing forest products like dry leaves/firewood in the house; having cattle in the house) or with a significant crude odds ratio (OR) in our dataset (no formal education, living in a mud house, regular tick sightings in the house, house< 500 m from monkey death site) were further analyzed in the multivariate model to calculate adjusted ORs with 95% CI.

### Ethical Statement

This study was conducted as part of a public health investigation to identify risk factors for a public health problem. All statutory permissions were obtained from the NCDC and the Karnataka Integrated Disease Surveillance Programme. Ethical approval was waived as the investigation was conducted in accordance with the applicable state and central government law (Epidemic Diseases Act no. 3, 1897). Strict data protection protocols reviewed by the NCDC were followed while collecting information from cases and controls. We obtained written informed consent from the study participants, treating physicians and program managers before in-person interviews and handling of clinical records.

## Results

### Descriptive Epidemiology of KFD Cases and Deaths

**FIGURE 1:**
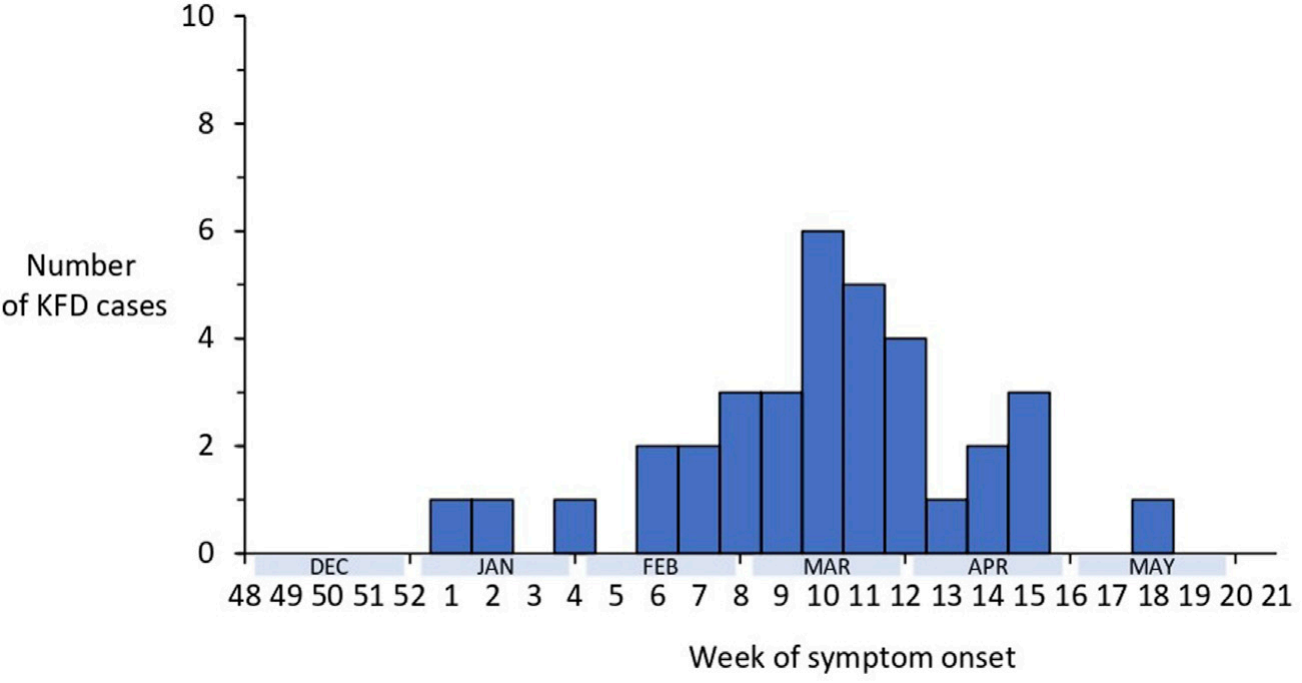
Distribution of laboratory-confirmed cases of Kyasanur Forest Disease by week of symptom onset, Shivamogga, Karnataka, January 2022-May 2022 (N = 35).

Analyzing the time trend of laboratory-confirmed KFD cases in the Shivamogga district over the last five KFD transmission seasons (2018–2022), we found that the number of cases in 2022 was below the outbreak threshold (z score = 0.17, p = 0.9). However, the proportion of cases from Thirthahalli taluk (n = 326, 54%) out of all cases from the Shivamogga district (N = 609) increased over the years (from 44% in 2018 to 86% in 2022). In total, 35 KFD cases were reported from the Shivamogga district between 1 January and 31 May 2022; of which 51% were men, with a median age of 46 (range = 4–75) years; and 37% were fully vaccinated (two primary doses plus booster) with the KFD vaccine. Cases started in January, and peaked in mid-March with the last case reported in May 2022 (Figure 1).

**FIGURE 2:**
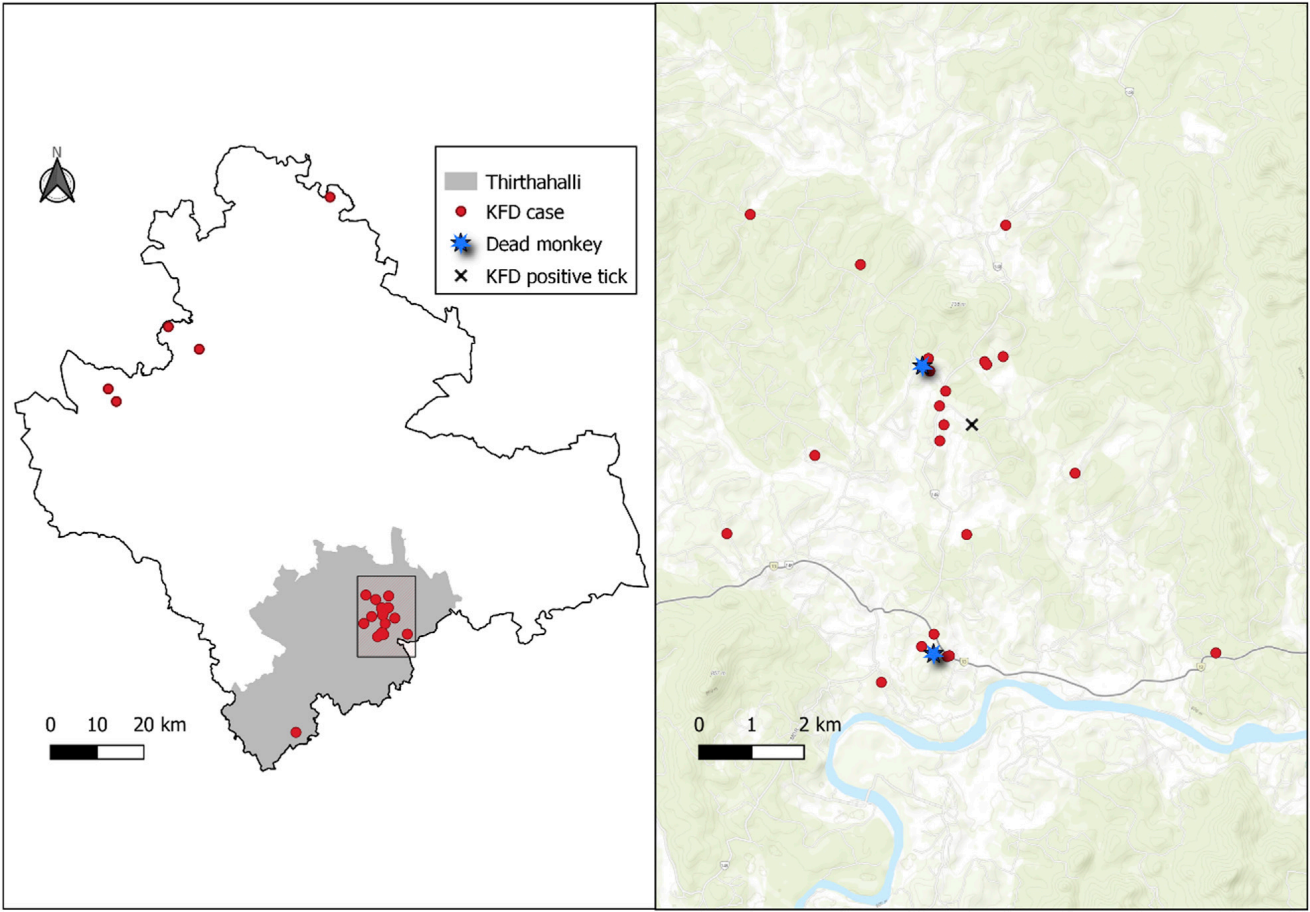
Left panel: Spot map (red dots) showing the geographical distribution of Kyasanur Forest Disease (KFD) cases (N = 35), Shivamogga, January 2022-May 2022. Right panel: Spot map showing the distribution of KFD cases (red dots) in Thirthahalli along with the site of spotting dead monkeys (blue star) and location of positive tick pool (black “X”). Thirthahalli, January 2022–May 2022.

A spot map showing the distribution of KFD cases in the Shivamogga district with a focus on the Thirthahalli cluster shows the clustering of cases around the reported monkey death area and the positive tick pool collection site (Figure 2).

There was one death (case fatality rate 3%). The patient was a 56-year-old male, unvaccinated for KFD, with no known co-morbidities, who developed a fever with a headache, tested positive for KFD on day 5 of illness and was admitted to a tertiary care hospital on the same day. He progressively developed seizures, hepatic encephalopathy, metabolic acidosis and acute renal failure during his hospital stay and died on day 12 of the illness. The patient’s house was adjacent to the forest (<50 m), but relatives did not report the patient visiting the forest in the 2 weeks prior to illness onset, nor did they report any monkey deaths within 500 m in the preceding 3 months.

#### Clinical Course

Of the 35 cases from the district, 30 were reported from Thirthahalli taluk with a median age of 52 (4–75) years, 52% of which were women and 44% were fully vaccinated. Of these, we interviewed 25 cases. We could not contact 5 cases. Among the 25 cases, common symptoms were sudden onset fever (96%), prostration (84%), body aches (80%) and headache (64%); complications included bleeding manifestations (40%), altered sensorium/seizures (8%) and hepatic encephalopathy (4%) (Table 1). All cases visited a primary care/secondary care health facility to seek treatment; cases that developed complications (80%) such as signs of hypotension, impending shock or hepatic involvement were referred to a tertiary care hospital. Seven cases (28%), reported not being involved in forest/plantation activities but reported working with dry leaves, firewood and cattle in the household. These cases included students, children and the elderly.

**TABLE 1:** Clinical characteristics (N = 25) and laboratory findings (n = 13), Kyasanur Forest Disease cases, Thirthahalli taluk, Shivamogga, Karnataka, Jan 2022 – May 2022.

Symptoms (N = 25)	n	(%)
Fever	24	(96)
Fatigue	21	(84)
Body aches	20	(80)
Headache	16	(64)
Chills	15	(60)
Redness of the eyes	12	(48)
Nausea	11	(44)
Loss of appetite	11	(44)
Vomiting	8	(32)
Hypersensitivity to light	8	(32)
Stomach pain	7	(28)
Loose stools	5	(20)
Facial swelling	3	(12)
Cough	2	(8)
Shortness of breath	2	(8)
**Complications (N = 25)**		
Bleeding diathesis	9	(36)
Hematemesis (Blood in vomit)	4	(15)
Petechial hemorrhages in skin/mucosa	2	(7)
Hematochezia (Blood in stool)	2	(7)
Epistaxis (Nosebleed)	1	(4)
Vaginal bleeding	1	(4)
Altered sensorium/seizures	2	(8)
Hepatic encephalopathy	1	(4)

Laboratory findings (n = 13)	Median	(Range)	Geometric mean	(Standard deviation)
Hemoglobin (g/dL)	13.1	(4.1–14.8)	11.7	(2.7)
Total leukocyte count (cells/µL)	2,100	(1,000–7,600)	2,377	(1,835)
Platelet count (cells/µL)	58,000	(31,000–1,95,000)	72,612	(49,061)
Liver Function Tests SGOT^a^(U/L)	195	(35–440)	173	(308)
SGPT^b^(U/L)	89.5	(27–260)	100	(136)
Kidney Function Tests: Blood Urea(mg/dL)	26	(17–27)	22.8	(5.5)
Serum Creatinine(mg/dL)	0.9	(0.8–0.9)	0.9	(0.06)
Uric Acid(mg/dL)	3.4	(2.4–4.0)	3.2	(0.8)
Random Blood Sugar(mg/dL)	142	(87–412)	193	(158)

^a^ SGOT: Serum glutamic-oxaloacetic transaminase.

^b^ SGPT: Serum glutamic-pyruvic transaminase.

We obtained case files of 13 patients (seven men, six women). In 77% of cases, bicytopenia (leukopenia with thrombocytopenia) was observed on admission with a median total leukocyte count of 2,100 cells/µL (1,000–7,600 cells/µL) and platelet count of 58,000 cells/µL (3,100–195,000 cells/µL) (Table 1).

### Case-Control Study

We included 25 cases and 90 controls from Thirthahalli taluk. Among 25 cases (median age = 52 years, women = 52%) and 90 controls (median age = 49 years, women = 54%), demographic factors were comparable (p-value >0.05) except for their educational level. Among the study participants, 44% of cases and 39% of controls were vaccinated with two doses of primary vaccine and an annual booster; and 20% of cases and 16% of controls were never vaccinated (Table 2).

**TABLE 2:** Socio-demographic characteristics of Kyasanur Forest Disease cases and controls, Thirthahalli taluk, Shivamogga, Karnataka, Jan 2022 – May 2022.

Socio-demographic characteristics		Cases (N = 25)	(%)	Control (N = 90)	(%)	*p*-value
Age (in years)	Median (range)	52	(4–75)	49	(15–85)	0.69
	Mean (SD)	46.9	(18.2)	48.3	(15.8)	
Sex	N (%) Men	12	(48)	41	(46)	0.82
Religion	Hindu	25	(100)	90	(100)	0.7
Caste^a^	Deprived	16	(64)	53	(59)	0.8
Occupation	Farm owner	4	(16)	27	(30)	0.2
	Farm Labourer	14	(56)	30	(33)	
	Student	2	(8)	7	(8)	
	Household dweller	4	(16)	21	(23)	
	Others	1	(4)	5	(6)	
Type of dwelling	Mud house	4	(16)	1	(1)	**0.007**
	Concrete house	21	(84)	89	(99)	
Educational status	No formal education	9	(36)	13	(14)	**0.01**
	Some formal education (Primary/High school/College)	16	(64)	77	(86)	
Addictions	Alcohol	2	(8)	6	(7)	0.4
	Tobacco	2	(8)	2	(2)	
Comorbidities	Diabetes	2	(8)	8	(9)	0.3
	Hypertension	4	(16)	18	(20)	
	Liver disease	0		0		
	Cancer	0		0		
	COVID	0		0		
	Others	1	(4)	4	(4)	
Vaccination status^b^	Two doses with booster	11	(44)	35	(39)	0.8
	Two doses only	5	(20)	17	(19)	
	One dose only	4	(16)	24	(27)	
	Never vaccinated	5	(20)	14	(16)	
Knowledge of KFD	Caused by Ticks	16	(64%)	53	(59%)	0.7
	Prevented by these					
	a. Vaccine	7	(28%)	18	(20%)	
	b. DMP/DEPA oil^c^	14	(56%)	42	(47%)	
	c. Wearing full-body clothing	3	(12%)	16	(18%)	
	d. Avoiding monkey carcass areas	0	(0%)	10	(11%)	
	e. Injecting cattle with ivermectin	0	(0%)	0	(0%)	
	f. Washing cattle regularly	1	(4%)	0	(0%)	
	g. Avoiding dry leaf collection	3	(12%)	12	(13%)	

^a^ Castes have been grouped into deprived/non-deprived according to the latest central government’s list of backward castes published by the National Commission for Backward Castes, 2015.

^b^ Vaccination status of cases was confirmed from health records. Vaccination status of controls was based on recall.

^c^ DMP: Dimethyl pthalate, DEPA: N,N-Diethyl Phenylacetamide.

Boldface indicates significance.

Both cases and controls had limited knowledge regarding preventive measures against KFD, with no significant difference in the knowledge between the groups. None of the cases and few of the controls (0% of cases, 11% of controls, p-value = 0.4) reported knowledge of acquiring the disease via exposure to dead/dying monkeys and the need to avoid the site of recent monkey death. Few (12% of cases, 18% of controls, p-value = 0.7) reported wearing full-body clothing or other protective clothing while working in forest/farmland to protect against tick bites (Table 2).

On bivariate analysis, living in a mud house [OR 17.0, 95% CI 1.8–159], residing within 500 m of a site where a dead monkey had been spotted in the previous 3 months [OR 6.8, 95% CI 1.7–26.4], and household tick exposure [OR 3.6, 95% CI 1.4–8.9] were significant risk factors for disease.

On multivariate analysis, the odds of acquiring KFD did not differ significantly between the vaccinated and unvaccinated study participants [aOR 1.5, 95% CI 0.5–4.6]. Residing within 500 m of a site where a dead monkey had been spotted in the previous 3 months [aOR 8.6, 95% CI 1.8–41.9] and household tick exposure [aOR 3.7, 95% CI 1.3–10.7] were significant independent risk factors for illness (Table 3).

**TABLE 3:** Odds Ratio (OR) and adjusted odds ratio (aOR) of disease among Kyasanur Forest Disease cases and controls by distribution of risk factors, Thirthahalli taluk, Shivamogga, Karnataka, Jan 2022 – May 2022.

Exposures	Cases (N = 25)	Controls (N = 90)	OR	(95% C.I.)	aOR^a^	(95% C.I.)
Living in a mud house	4	1	17.0	(1.8–159)	Excluded^b^	
House <500 m of monkey death site (in the previous 3 months)	6	4	6.8	(1.7–26.4)	**8.6**	(1.8–41.9)
Low socioeconomic status^c^	4	4	4.1	(0.9–17.7)	4.5	(0.7–29.3)
Household tick exposure^d^ (in the previous 3 months)	13	20	3.6	(1.4–8.9)	**3.7**	(1.3–10.7)
No formal education	9	13	3.3	(1.2–9.1)	Excluded^e^	
House located <50 m from the forest tree line	18	42	2.9	(1.1–7.7)	2.7	(0.9–8.0)
Regular tick sightings in the house	13	25	2.8	(1.1–6.9)	Excluded^e^	
Either dry leaves or firewood stored in the house	23	73	2.7	(0.6–12.5)	Excluded^f^	
Involved in the handling of forest products in the previous 3 months	16	41	2.1	(0.8–5.3)	Excluded^f^	
Dry leaves stored in the house	21	65	2.0	(0.6–6.4)	Excluded^f^	
Two doses of KFD vaccine with/without booster	16	52	1.3	(0.5–3.2)	1.5	(0.5–4.6)
Having cattle in the house	6	19	1.2	(0.4–3.4)	Excluded^f^	
Handling of forest products in the household (without going to the forest)	7	32	0.7	(0.2–1.8)	Excluded^f^	

Boldface indicates significance.

a We adjusted for all other exposures that conferred a high risk of acquiring the disease according to the unadjusted OR.

b Excluded from multivariate analysis due to very wide 95% CI, in bivariate analysis.

c Low socio-economic status was defined as any two of the following: no formal education; residing in a non-cemented house; not owning cattle.

d Household tick exposure was defined as ‘tick sightings AND, storage of dry leaves/firewood in the house.

e Excluded from multivariate analysis due to statistically significant correlation with low socioeconomic status variable and household tick exposure.

f Excluded from multivariate analysis as the variable did not meet the statistical inclusion criterion.

### Assessment of the KFD Prevention and Control Program (in the 2021–2022 KFD Transmission Season)

#### Surveillance of Human Cases, Tick Pools, and Monkey Deaths

For human cases (active and passive), intensified active surveillance was conducted within a 5 Km radius following evidence of KFDV transmission, with a transition to daily passive reporting from monthly reporting during the outbreak season, as recommended in the operational guidelines.

One tick pool from the Thirthahalli block tested on 28 February 2022 was positive for the KFD virus out of 79 tick pools collected from January to May 2022 in Thirthahalli for virus identification in ticks to map hotspots.

Shivamogga reported 48 monkey deaths, of which 11 (23%) were reported in Thirthahalli. Geocoordinates were available for two (18%) of the 11 monkey carcasses reported in Thirthahalli, both of which were necropsied and reported negative for KFDV. State officials reported challenges in performing all monkey necropsies, and not receiving timely necropsy reports to take appropriate public health action for decontamination of monkey death sites.

Prevention and control activities included vaccination in hotspots, Information, education and communication (IEC), and diagnostic and treatment facilities/transportation.

For an eligible population of 53,264 in Thirthahalli, 21,586 doses (40%) including a booster and a second dose of the KFD vaccine were administered. Maintenance of the vaccine cold chain at the CHC and PHC levels was challenging during prolonged power outages. Staff also reported a shortage of vaccines for the upcoming season.

During the study period, 10 advocacy campaigns, 12 loudspeaker announcements, 73 awareness events in schools/colleges and 45 group discussions (at the village level) were conducted by the health department regarding KFD, which was satisfactory as per the operational guidelines for KFD.

Once a patient visited the health facility, they were tested and test results were communicated within a median of 2 (range = 0–7) days. The taluk hospital had a dedicated ward with 20 beds and round-the-clock nursing support, and the cost of treatment and transportation was borne by the state government (Table 4).

**TABLE 4:** Review of Kyasanur Forest Disease (KFD) prevention and control activities in Thirthahalli taluk, Shivamogga against its annual program implementation plan, June 2021 to May 2022.

Activity	Timeline (expected)	Status for 2021–2022	Records reviewed and remarks (satisfactory/unsatisfactory)
Active surveillance of human cases after detection of KFD cases in the area	Jan–Feb	Present	Satisfactory Surveillance activities and outbreak investigation reports reviewed from Jan to May 2022 State and district rapid response team (RRT) constituted Cumulative monthly reporting data for CHC Kannangi show intensified fever surveillance, tick surveillance and increased testing of suspected KFD cases
Tick surveillance	Oct. onward	Done	Satisfactory Records assessed at VDL show 324 tick pools tested (201 in 2021 and 123 in 2022) in Shivamogga One tick pool tested positive for KFD virus out of 79 tick pools collected in Thirthahalli
Monkey death: mapping/carcass disposal/necropsy	Oct. onward		Unsatisfactory Shivamogga reported 44 monkey deaths, of which 11 (25%) were reported in Thirthahalli Geolocation mapping and necropsy of monkey deaths were conducted for 2/11 (18%) deaths occurring in Thirthahalli taluk; both necropsies reported as KFDV-negative State officials report challenges in conducting monkey necropsies and getting timely reports of samples sent to a reference laboratory
Passive surveillance Transition from monthly to daily reporting during KFD transmission season	Oct–June	Done	Satisfactory Records assessed show intensified daily reporting during transmission season
Outbreak response activities in response to human/monkey/tick-positive	Dec-June		Satisfactory In response to the human-positive case, surrounding villages within a 5 km radius were vaccinated State RRT visited cases including death cases and these records were reviewed
Tick control measures	Oct. onward	Done with delay	Unsatisfactory Records assessed from the veterinary department Control measures implemented in villages reporting positive human/monkey/tick cases in the previous season Control measures not implemented in all villages within a 5 km radius due to lack of funds Delay between reporting a case and procuring medications was 2–3 months, which delayed control efforts
KFD Vaccination (a) Mass KFD vaccination in high-risk areas: first dose (b) Mass KFD vaccination in high-risk areas: completion of second dose	(a) June–July (b) Aug	Done in 2021 Not done in 2022	Unsatisfactory In the KFD transmission season of 2021–2022, for an eligible population of 53,264 in Thirthahalli, 21,586 doses (40%) including a booster and second dose of the KFD vaccine were administered Vaccine reached PHC near the expiration date (<1 month before expiration) Maintaining the cold chain for vaccines an issue due to irregular electricity supply KFD Vaccine hesitancy/refusal due to COVID vaccine drive (reluctance to take two vaccines) Vaccine not available in the season 2022–2023
Diagnosis of cases Median time (in days) for each step of the diagnostic process	Dec. onward		Satisfactory Records of 25 cases from Thirthahalli reviewed and cross-checked with hospital records From the date of the health facility visit to the KFD test: median days (0–7) From the date of the test to the test result: median days = 2 (0–6) From test result to hospitalization: median days = 0.5 (0–1)
Treatment of cases Facilities available in secondary care hospital/referral to tertiary care facility/patient transport	Dec. onward		Satisfactory The KFD ward in the Taluk hospital, Thirthahalli was observed during a facility visit The ward was equipped with 20 beds and 24-h nursing support Patient management/categorization protocols were available Physicians were aware of protocol steps For patients needing referral, transport was arranged from the hospital to the referral center The cost of treatment at the referral center was borne by the state government
Information Education and Communication measures (IEC) Distribution of DEC Control efforts	Oct. onward		Satisfactory From Jan-May 2022, CHC Kannangi area conducted 10 advocacy campaigns, 12 miking events, 73 awareness events in schools/colleges and 45 group discussions In total, 3,100 tick repellents (DMP/DEPA) were distributed Two (100%) hotspots were sprayed with insecticide after monkey deaths

Source: List of activities and their expected timeline from Annexure XXII, p 93, Operational manual KFD, department of health and family welfare, Government of Karnataka, 2020.

## Discussion

There was a laboratory-confirmed cluster of KFD cases in Thirthahalli taluk from January to May 2022 with cases exhibiting symptoms, complications, and laboratory findings consistent with published literature [9–12]. Vaccination coverage was low, both in cases and controls with age groups not targeted for vaccination also being affected, as reported in past outbreaks by other studies [9, 12–14]. This study indicates that the odds of developing the disease did not differ significantly between the vaccinated and unvaccinated study participants. This result is similar to that of Kasabi et al who conducted a matched case-control study in the Shimoga district in 2011–2012 and concluded that two doses of the vaccine did not protect the at-risk population from developing KFD [13].

This study identified residing in proximity of dead monkey sites as a significant risk factor for acquiring KFD; this evidence is in agreement with other studies reporting KFD outbreaks in areas reporting monkey deaths [15, 16], and in populations handling dead/dying monkeys [3]. However, unlike previous studies, none of our cases reported handling a dead monkey, therefore our results indicate possible indirect exposure of cases to infected tick vectors from monkey carcass sites. We recommend a review of monkey carcass disposal procedures, timely necropsy and sampling of all monkey deaths for the presence of KFDV, and intensified communication and fever surveillance in surrounding areas to prevent indirect exposure of residents to infected ticks from monkey carcasses.

Limited knowledge among our study participants regarding preventive measures and the need to avoid the site of recent monkey deaths may also put them at increased risk of acquiring the disease, as reported by Asaaga et al [17]. This may be related to their socioeconomic status, as significantly more cases than controls had not completed any formal education, disproportionately affecting their access to the disease knowledge available in newspapers and disease information leaflets distributed as part of the KFD prevention and control program. To reach the most vulnerable population and address this knowledge gap, governments should prioritize risk communication in the form of announcements in the local language, folk songs, and street plays over written material.

Although occupational exposure to forests has been implicated as a risk factor for KFD(13), in this study, there were cases who did not visit the forest but handled cattle and forest products in the household. Murhekar et al have reported similar findings and suggest that close contact with cattle that graze in infected forests, and with forest products from an infected forest could transport infected ticks into the household, exposing residents not otherwise involved in forest activities, to the disease [18]. These at-risk populations should be included in risk communication including the application of indoor tick repellents while handling dry leaves/firewood/cattle during the outbreak season.

A review of the KFD prevention and control program at Thirthahalli revealed adequate diagnostic and treatment measures once a human case was detected but indicated possible gaps in preventive measures, including vaccination coverage, monkey necropsies, and tick control measures. Addressing challenges in monkey necropsies and ensuring timely necropsy reports to relevant stakeholders will help the program map KFD disease hotspots and conduct timely site decontamination. Similarly, sharing tick pool surveillance reports will facilitate better planning for tick control activities. In addition, incorporating machine learning approaches, along with the use of weather and event-based surveillance data, can help the program predict KFD outbreaks in existing and new foci to plan preventive measures [19].

This study has at least three limitations. We anticipate recall bias among study participants regarding exposures and vaccination history especially recall of the primary vaccination schedule taken a few years earlier. This could have impacted the strength of the association between vaccination and contracting the disease. We tried to limit this by reviewing vaccination records, whenever possible. Our controls were recruited based on their history of no fever with myalgia in the previous 6 months. We could not recruit laboratory-negative KFD controls due to logistical issues. This could have reduced the strength of the association of the exposures by inadvertently recruiting subclinical cases as controls. We were only able to enroll the available cases within the study period, which may result in low study power. A longer study duration may be needed to obtain adequate cases.

### Conclusion

This study underlines the importance of a holistic One health approach involving interdisciplinary stakeholders from human, animal and environmental health in implementing effective surveillance and control strategies for emerging and re-emerging zoonotic diseases like KFD. In our study vaccination did not offer protection against KFD. We recommend further studies with a bigger sample size to determine vaccine protection against KFD. Based on our results, we recommend evaluating current monkey carcass disposal and necropsy procedures, increasing awareness of the role of monkey carcasses in disease transmission and effective tick control measures around monkey carcass sites. Intensified behavior change communication should be undertaken in at-risk communities with a focus on tick protective measures including wearing full body clothing, applying DMP/DEPA oil when entering a forest/plantation or handling forest produce at home, avoiding monkey carcass areas and tick removal after coming back from the forest.

## References

[1] Holbrook MR. Kyasanur Forest Disease. Antivir Res. (2012) 96(3):353–62. 10.1016/j.antiviral.2012.10.005 23110991 PMC3513490

[2] Shah SZ Jabbar B Ahmed N Rehman A Nasir H Nadeem S Epidemiology, Pathogenesis, and Control of a Tick-Borne Disease- Kyasanur Forest Disease: Current Status and Future Directions. Front Cell Infect Microbiol (2018) 8:149. 10.3389/fcimb.2018.00149 29868505 PMC5954086

[3] Sadanandane C Elango A Marja N Sasidharan PV Raju KHK Jambulingam P. An Outbreak of Kyasanur Forest Disease in the Wayanad and Malappuram Districts of Kerala, India. Ticks Tick-borne Dis (2017) 8(1):25–30. 10.1016/j.ttbdis.2016.09.010 27692988

[4] Government of Karnataka. Directorate of Health and Family Welfare Services. Operational Guidelines. Kyasanur Forest Disease, 2020. Chapter 5, Operational guidelines for surveillance; p. 34–49.

[5] Pattnaik P. Kyasanur Forest Disease: An Epidemiological View in India. Rev Med Virol (2006) 16(3):151–65. 10.1002/rmv.495 16710839

[6] Chakraborty S Andrade FCD Ghosh S Uelmen J Ruiz MO. Historical Expansion of Kyasanur Forest Disease in India from 1957 to 2017: A Retrospective Analysis. GeoHealth (2019) 3(2):44–55. 10.1029/2018GH000164 32159030 PMC7007137

[7] Yadav PD Sahay RR Mourya DT. Detection of Kyasanur Forest Disease in Newer Areas of Sindhudurg District of Maharashtra State. Indian J Med Res (2018) 148(4):453–5. 10.4103/ijmr.IJMR_1292_17 30666009 PMC6362715

[8] Patil DY Yadav PD Shete AM Nuchina J Meti R Bhattad D Occupational Exposure of Cashew Nut Workers to Kyasanur Forest Disease in Goa, India. Int J Infect Dis IJID Off Publ Int Soc Infect Dis (2017) 61:67–9. 10.1016/j.ijid.2017.06.004 28627428

[9] Kasabi GS Murhekar MV Sandhya VK Raghunandan R Kiran SK Channabasappa GH Coverage and Effectiveness of Kyasanur Forest Disease (KFD) Vaccine in Karnataka, South India, 2005–10. Plos Negl Trop Dis (2013) 7(1):e2025. 10.1371/journal.pntd.0002025 23359421 PMC3554520

[10] Work TH Trapido H Murthy DPN Rao RL Bhatt PN Kulkarni KG. Kyasanur Forest Disease. III. A Preliminary Report on the Nature of the Infection and Clinical Manifestations in Human Beings. Indian J Med Sci (1957) 11(8):619–45. 13474777

[11] Gladson V Moosan H Mathew S P D. Clinical and Laboratory Diagnostic Features of Kyasanur Forest Disease: A Study from Wayanad, South India. Cureus (2021) 13(12):e20194. 10.7759/cureus.20194 35004016 PMC8728626

[12] Kiran SK Pasi A Kumar S Kasabi GS Gujjarappa P Shrivastava A Kyasanur Forest Disease Outbreak and Vaccination Strategy, Shimoga District, India, 2013–2014. Emerg Infect Dis (2015) 21(1):146–9. 10.3201/eid2101.141227 25531141 PMC4285264

[13] Kasabi GS Murhekar MV Yadav PD Raghunandan R Kiran SK Sandhya VK Kyasanur Forest Disease, India, 2011-2012. Emerg Infect Dis (2013) 19(2):278–81. 10.3201/eid1902.120544 23343570 PMC3559039

[14] Bhat P S JH Raju MK Sooda S K P Kumar R. Kyasanur Forest Disease, Is Our Surveillance System Healthy to Prevent a Larger Outbreak? A Mixed-Method Study, Shivamogga, Karnataka, India: 2019. Int J Infect Dis (2021) 110:S50–61. 10.1016/j.ijid.2021.07.076 34416404

[15] Mourya DT Yadav PD Sandhya K Reddy S. Spread of Kyasanur Forest Disease, Bandipur Tiger Reserve, India, 2012–2013. Emerg Infect Dis (2013) 19(9):1540–1. 10.3201/eid1909.121884 23977946 PMC3810911

[16] Chakraborty S Sander WE Allan BF Andrade FCD. Retrospective Study of Kyasanur Forest Disease and Deaths Among Nonhuman Primates, India, 1957–2020. Emerg Infect Dis (2021) 27(7):1969–73. 10.3201/eid2707.210463 34152964 PMC8237885

[17] Asaaga FA Rahman M Kalegowda SD Mathapati J Savanur I Srinivas PN ‘None of My Ancestors Ever Discussed This Disease before!’ How Disease Information Shapes Adaptive Capacity of Marginalised Rural Populations in India. 15 (2021).Plos Negl Trop Dis 3 e0009265. 10.1371/journal.pntd.0009265 33705400 PMC7987196

[18] Murhekar MV Kasabi GS Mehendale SM Mourya DT Yadav PD Tandale BV. On the Transmission Pattern of Kyasanur Forest Disease (KFD) in India. Infect Dis Poverty (2015) 4:37. 10.1186/s40249-015-0066-9 26286631 PMC4545326

[19] Keshavamurthy R Charles LE. Predicting Kyasanur Forest Disease in Resource-Limited Settings Using Event-Based Surveillance and Transfer Learning. Sci Rep (2023) 13(1):11067. 10.1038/s41598-023-38074-0 37422454 PMC10329696

